# Strategies to Overcome Heparins’ Low Oral Bioavailability

**DOI:** 10.3390/ph9030037

**Published:** 2016-06-29

**Authors:** Ana Rita Neves, Marta Correia-da-Silva, Emília Sousa, Madalena Pinto

**Affiliations:** 1 Organic Chemistry and Pharmaceutical Laboratory, Department of Chemical Sciences, Faculty of Pharmacy, University of Porto, Rua Jorge Viterbo Ferreira 228, 4050-313 Porto, Portugal; anarcneves92@gmail.com (A.R.N.); m_correiadasilva@ff.up.pt (M.C.-S.); madalena@ff.up.pt (M.P.); 2 Interdisciplinary Centre of Marine and Environmental Research (CIIMAR/CIMAR), University of Porto, Rua dos Bragas 289, 4050-123 Porto, Portugal

**Keywords:** heparin, anticoagulant, oral bioavailability

## Abstract

Even after a century, heparin is still the most effective anticoagulant available with few side effects. The poor oral absorption of heparins triggered the search for strategies to achieve oral bioavailability since this route has evident advantages over parenteral administration. Several approaches emerged, such as conjugation of heparins with bile acids and lipids, formulation with penetration enhancers, and encapsulation of heparins in micro and nanoparticles. Some of these strategies appear to have potential as good delivery systems to overcome heparin’s low oral bioavailability. Nevertheless, none have reached the market yet. Overall, this review aims to provide insights regarding the oral bioavailability of heparin.

## 1. Introduction

Heparin has been one of the most effective and widely used drugs of the past century. Discovery of heparin dates back to 1916, when isolated blood fractions that were able to clot blood were identified by Jay McLean, a second-year medical student working under the direction of William Howell [1].

Chemically, heparin is a mixture of highly sulfated glycosaminoglycans with molecular weight around 15 kDa. The presence of sulfate and carboxylic acid groups in the structure of heparin makes it one of the most negatively charged biological macromolecules in nature [2,3,4]. In physiological conditions, ionization of sulfate and carboxylic acid groups takes place, which attracts positively charged counter ions, more commonly sodium, to form a heparin salt.

Low molecular weight heparins (LMWHs), such as unfractioned heparin (UFH), are also mixtures of glycosaminoglycans but with an average molecular weight of about 5 kDa [5]. LMWHs derive from UFH by chemical or enzymatic depolymerization and have a more predictable pharmacokinetic profile [6] and a slightly different mechanism of action.

**Figure 1:**
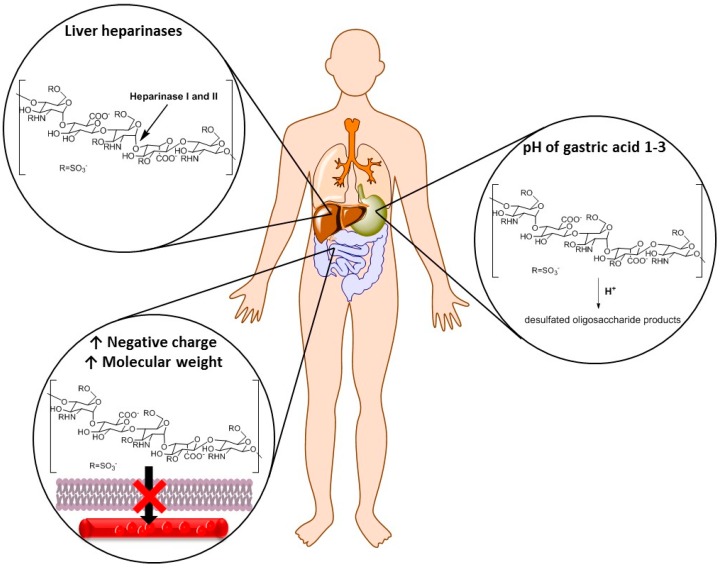
Structural features of heparins that limit their oral bioavailability. UFH—Unfractioned heparin.

A unique pentasaccharide sequence present in both UFH and LMHWs is essential for their anticoagulant activity. UFH binds to antithrombin III (ATIII) that undergoes a conformational change and becomes activated as an inhibitor of thrombin and factor Xa (FXa) while LMWHs are shorter and show limited inhibition of thrombin [5]. Because the bioavailability of subcutaneous UFH is lower than that of intravenous UFH, UFH is preferably administered intravenously to avoid the administration of large doses of subcutaneous UFH [7]. Bioavailability of subcutaneous LMWHs is higher than that of subcutaneous UFH, so LMWHs are administered subcutaneously [5]. However, none exhibit oral bioavailability due to their highly negative charge, large molecular weight [8], and rapid metabolism in gastrointestinal (GI) tract (Figure 1) [9]. The rapid metabolism in the GI tract is mainly due to low stability at low pH [10] and enzymatic degradation by intestinal microflora [11,12]. Additionally, after GI absorption heparins undergo high first pass metabolism by heparinase in the liver [13].

**Figure 2:**
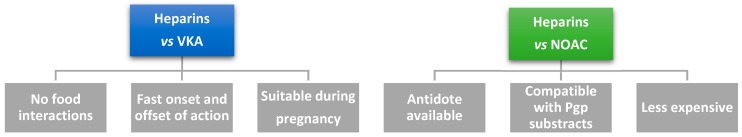
Advantages of heparins over vitamin K antagonists (VKA) and new oral anticoagulants (NOACs) drugs.

Parenteral administration of heparins is a costly and invasive method that leads to low patient adherence. On the other hand, vitamin K antagonists (VKA), orally active anticoagulants, suffer from a number of limitations, namely high food and drug interactions, long onset/offset of action, and need of regular monitoring by blood tests. New oral anticoagulants were recently introduced in the market that overcome some of those VKA limitations, namely dabigatran (direct thrombin inhibitor), rivaroxaban, apixaban, and edoxaban (FXa inhibitors), but there are some hesitations about their wide use in the treatment of thromboembolic diseases, namely the absence of an antidote and extensive drug interaction with P-glycoprotein (P-gp) substrates [14]. In general, heparins have several advantages over VKA and new oral anticoagulants (Figure 2) which makes them the most effective anticoagulants available. Furthermore, all these orally active anticoagulants lack the polypharmacological actions of heparins which are thought to be involved beyond the coagulation cascade [15], like antimetastatic [16] and anti-inflammatory activities [17].

The development of a non-invasive delivery of heparin is undoubtedly an unmet clinical need. Therefore, several strategies that promote the absorption of heparins in GI tract have been investigated in the last decades.

Although the oral route is by far the most desirable, it is important to emphasize that there also have been attempts to achieve bioavailability of heparins through other routes, such as nasal [18], pulmonary [19], and transdermal [20].

This review focus on (physico-)chemical-biological and technological strategies that are expected to improve oral bioavailability of heparins, namely the increase of lipophilicity, target receptor-mediated endocytosis, modifications of the tight junctions, enhancement of cell permeabilization, and protection against acidic pH of the stomach and GI metabolism.

## 2. Heparin Conjugates

Following a drug conjugate strategy, heparins have been covalently bond to other molecules in order to achieve oral bioavailability in one of two ways: increasing lipophilicity and permeability or enabling absorption via transporter proteins [21].

**Figure 3:**
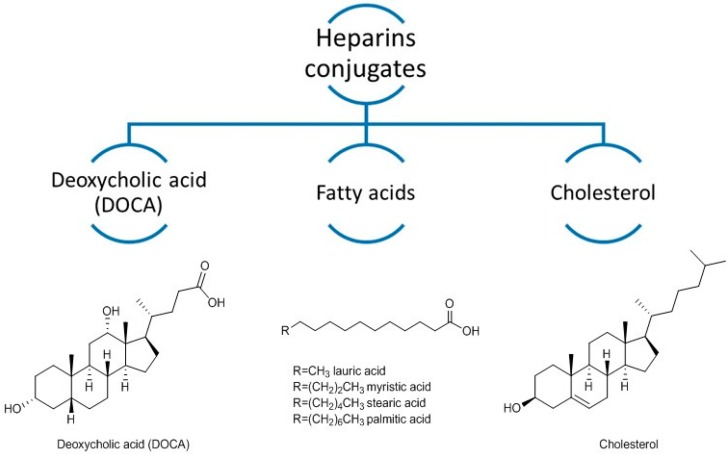
Chemical structure of deoxycholic acid (DOCA), fatty acids, and cholesterol.

Lipids and deoxycholic acid (DOCA) (Figure 3) have been proven to be suitable choices for conjugation with heparins [22,23,24,25,26,27,28,29,30,31,32,33,34]. As they are naturally occurring substances, the administration of these conjugates may involve few toxic effects [32]. Conjugates of heparin with palmitic acid, lauric acid, and cholesterol (Figure 3) were reported [32]. Absorption of these heparin-lipids conjugates increased in rats, probably due to their higher lipophilicity. Nevertheless, heparin-cholesterol and heparin-fatty acids conjugates showed lower effect in clotting times than heparin-DOCA conjugates [32]. DOCA is a secondary bile acid produced from cholesterol and one of its functions is to stimulate intestinal lipids absorption [35].

**Figure 4:**
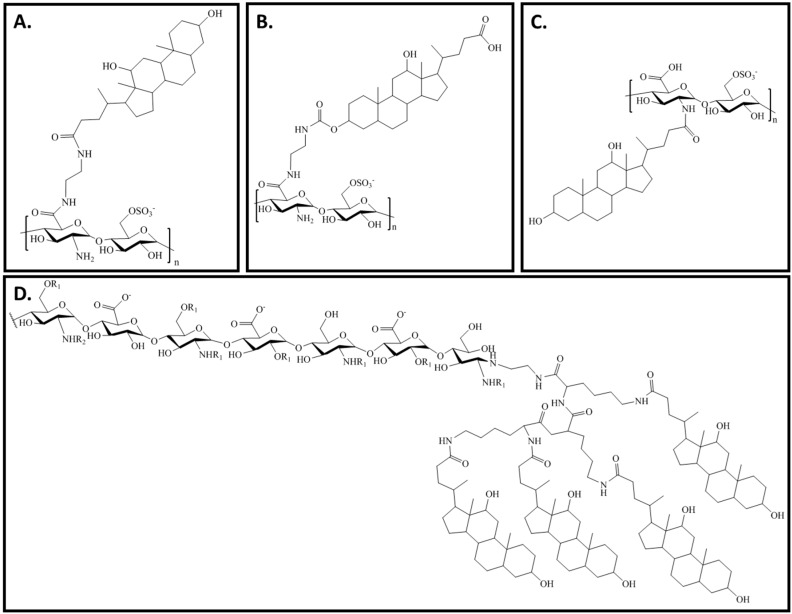
Structure of different heparins-DOCA conjugates. (**A**) Heparin-DOCA conjugate in which both carboxylic groups of heparin and DOCA were conjugated through a linker [22,23,24,30,31,33]; (**B**) Heparin-DOCA conjugate in which a carboxylic group of heparin and a hydroxyl group of DOCA were conjugated through a linker [29]; (**C**) Heparin-DOCA conjugate in which an amine group of heparin and a carboxylic group of DOCA were conjugated without a linker [25,26,27,28,32]; (**D**) Heparin-tetrameric DOCA conjugate [37].

DOCA is absorbed in the intestinal membrane through apical sodium bile acid transporters and this mechanism of absorption was also hypothesized to increase the transcellular absorption of heparins [22]. Numerous studies have proven that heparins-DOCA conjugates increased oral bioavailability of heparins by increasing their intestinal absorption (Figure 4A–C) [23,24,26,27,32]. DOCA was conjugated with heparins using different synthetic approaches, all of them involving the formation of one amide bond (Figure 4A–D). Some conjugates involved the carboxylic groups of heparin to conjugate with DOCA (Figure 4A,B) and others the amine groups (Figure 4C). Lee et al. carried out histological examination of GI tissue membrane after oral administration of heparin-DOCA and showed no damage on microvilli and cell layer [27,31,32]. These results proved that the increased absorption of heparins conjugates was not caused by the disruption of the GI epithelium.

One problem associated with the use of DOCA is that the conjugate with heparins forms micelles in water and DOCA is located within the core of micelles. As a consequence, lower interaction of DOCA with apical sodium bile acid transporters occurs, limiting the absorption of the conjugate. Thus, the bioavailability of heparin-DOCA conjugates was improved through the formulation with dimethylsulfoxide (DMSO) as a solubilizer [22,28,30,31]. DMSO is known to interfere with lipid membranes [36]. The absorption of heparin-DOCA with DMSO was three times higher compared with heparin-DOCA conjugate alone. Later, Kim et al. developed a tricaprylin microemulsion to dissolve heparin-DOCA conjugate [34]. Microemulsions are thermodynamically stable, liquid mixtures of oil, water, surfactant and co-surfactant that are used in pharmaceutical science to improve the solubility of drugs (amphiphilic or lipophilic). Tricaprylin is a medium-chain triglyceride marketed as a medical food for the clinical dietary management of Alzheimer’s disease. Heparin-DOCA conjugate in water was mixed with tricaprylin, polyoxyethylene 20 sorbitan monooleate (Tween 80) and sorbitan monolaurate (Span 20). Heparin-DOCA conjugate was dissolved in a microemulsion with tricaprylic and the absorption efficiency of the heparin conjugate was increased indicating that solubilization of heparin-DOCA conjugate is important for the absorption in the intestine.

A recent study reported the absorption of a heparin-tetrameric DOCA conjugate (Figure 4D) with a remarkable systemic anticoagulant activity and high oral bioavailability of 33.5% and 19.9% in rats and monkeys, respectively [37]. This study showed a functional transformation of apical sodium bile acid transporters upon interaction with tetrameric DOCA derivative, which led to receptor-mediated endocytosis [38]. 

Conventional anticancer drugs are toxic, and research is focus in the development of more specific drugs that target only molecular abnormalities specific to cancer cells. The benefits of heparin in cancer are both dependent and independent from its anticoagulant activities [39]. A LMWH-DOCA conjugate (Figure 4A) was also tested for antimetastatic [40] and antiangiogenic [41,42] activities. These studies reported for the first time the antitumor effect of an orally active heparin and confirmed that it inhibits tumor angiogenesis. The co-administration of LMWH-DOCA conjugate and docetaxel, a drug widely used in the treatment of several human cancers, showed a higher tumor growth inhibition compared to LMWH-DOCA conjugate or docetaxel alone [42]. Considering that DOCA has low solubility in water, LMWHs were conjugated with taurocholic acid and docetaxel [43,44]. Oral bioavailability and synergistic effects of heparin and docetaxel were observed in this conjugate. Recently, a conjugate of LMWH with taurocholic acid and tetrameric DOCA was prepared and an increased oral bioavailability and promising angiogenesis inhibition were observed [45]. The same conjugate was co-administered with deoxycholyl-l-lysyl-methylester and an increased oral bioavailability was also observed [46].

However, there are a number of bile acids available, with different affinities to apical sodium bile acid transporters. In a study considering the effect of different bile acids on the uptake of taurocholate assay [47], chenodeoxycholic acid was the most efficient, followed by DOCA, cholic acid, ursodeoxycholic acid, and litocholic acid. Therefore, it would be interesting to study the conjugation of heparin with other bile acids, namely with chenodeoxycholic acid.

Conjugation with several bile acids, including DOCA, was successfully performed to improve oral bioavailability of other drugs, namely acyclovir [47] and insulin [48] but none have yet entered clinical trials. Consequently, conjugation of heparins with bile acids seems a valuable strategy to improve oral bioavailability of heparins. However, this strategy will not protect heparins from degradation in the stomach, intestine, and liver. On the other hand, there are some studies indicating that an increased colonic exposure to bile acids could play a role in the development of cancer, since higher fecal concentrations of bile acids were found in populations with a high incidence of colorectal cancer [49,50,51], and the levels of exposure to bile acids must be considered in the development of heparin-bile acids conjugates.

## 3. Co-Administration with Penetration Enhancers

In this section, penetration enhancers that have already been used to improve UFH and LMWHs absorption is revised. The weak association between penetration enhancers and drugs allows the spontaneous release of the drug into the circulation [52]. However, in contrast to drug conjugate strategy, some dissociation before absorption cannot be excluded.

**Figure 5:**
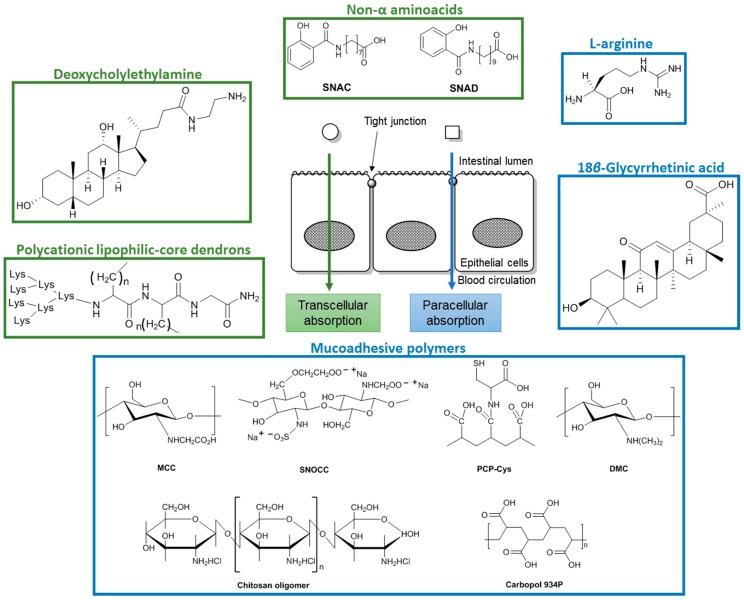
Chemical structure of penetration enhancers that increase oral bioavailability of heparins. MCC—Mono-*N*-carboxymethyl chitosan; SNOCC: *N*,*O*-Carboxymethyl chitosan; PCP-Cys: Polycarbophil-cystein; DMC—*N*,*N*-Dimethyl chitosan; SNAD—Sodium *N*-[10-(2-hydroxybenzoyl) amino decanoate; SNAC: Sodium *N*-(8-[2-hydroxybenzoyl] amino) caprylate.

Penetration enhancers improve absorption of drugs in the GI tract either through paracellular absorption, by opening the tight junctions between adjacent cells, or through transcellular absorption, by increasing the lipophilic properties of the drug (Figure 5) [21,53]. Many of the penetration enhancers that act through paracellular absorption are described as causing cytotoxicity or membrane damage [54].

### 3.1. Paracellular Absorption

l-Arginine (Figure 5), a nonessential amino acid, is the principal physiological precursor of nitric oxide and plays a versatile role in the GI tract physiology. Intestinal absorption of a LMWH (ardeparin) was increased in the presence of l-arginine in Caco-2 cells [55]. In vivo studies in rats showed that absorption of ardeparin in the presence of l-arginine is higher in the colon and jejunum, and for 250 mg of l-arginine it was observed an increased anti-FXa activity compared with ardeparin alone. A sodium bicarbonate solution was administered with the LMWH/l-arginine formulation to increase the acidic pH of the stomach and to avoid degradation. Anti-FXa activity was used for estimating plasma levels of LMWH and a significant increase in plasma anti-FXa levels within 90 min to 0.26 IU/mL was observed after administration of ardeparin/l-arginine formulation (~2 fold compared with the control, 0.13 IU/mL). The effect of several concentrations of l-arginine on cell viability was investigated through mitochondrial dehydrogenase activity and there was no significant variation compared to the negative control [55].

18β-Glycyrrhetinic acid (GA, Figure 5) is a pentacyclic triterpenoid amyrin derivative obtained from the hydrolysis of glycyrrhizic acid, naturally present in the roots of the plant *Glycyrrhiza glabra*. GA was tested as a penetration enhancer in order to increase the intestinal absorption of LMWHs [56]. A sodium bicarbonate solution was also administered with the LMWH/GA formulation. Absorption of LMWHs was increased both in vitro and in vivo after co-administration with GA. Anti-FXa activity was used for estimating plasma levels of LMWH and a significant increase in plasma anti-FXa levels within 4 h to 0.28 IU/mL was observed after administration of GA (50 μg/kg) with ardeparin (1200 IU/kg). After exposure to GA, no significant toxicity was found in Caco-2 cells monolayers, at relatively low concentrations. The authors hypothesised that GA acts by increasing paracellular absorption of LMWHs [57]. This study was conducted by Hisamitsu Pharmaceutical Co., Inc. (Saga Prefecture, Japan); however, at the moment, GA seems to be applied only on transdermal formulations with pain relief drugs [58]. Recently, anticoagulant properties through uncompetitive inhibition of FXa were described for GA [59]. Therefore, co-administration of heparin with GA seems an interesting strategy that could increase both intestinal absorption and FXa inhibition. However, higher bleeding risk should be considered.

Mucoadhesive polymers increase the residence and the contact times of the drug with mucous membranes leading to a longer-lasting therapeutic effect [60]. These polymers are not absorbed in the GI tract due to their high molecular weight. Three mucoadhesive polymers, chitosan derivatives, poly(acrylates) and thiolated polymers, were described to increase the absorption of heparins in GI tract [61,62,63,64,65,66].

Chitosan is a polysaccharide with mucoadhesive properties comprising glucosamine and *N*-acetylglucosamine subunits [54]. In vitro studies have shown that chitosan opens epithelial tight junctions in a concentration- and pH-dependent way [54]. However, chitosan was incompatible with LMWHs [67] and chitosan derivatives were prepared [61,62]. Mono-*N*-carboxymethyl chitosan (MCC, Figure 5), a polyampholyte chitosan derivative was shown to increase in vitro paracellular absorption of LMWHs in Caco-2 cell monolayers [61]. The in vivo intestinal absorption of LMWHs was also increased with MCC in rats. Anti-FXa activity was used for estimating plasma levels of LMWH and therapeutic LMWH levels (0.3–0.7 anti-FXa U/mL) were reached 3 h after administration with MCC. A sulfonate derivative of *N*,*O*-carboxymethyl chitosan (SNOCC, Figure 5) increased the permeation and absorption of LMWHs both in vitro (Caco-2 cell monolayers) and in vivo (rats) after intraduodenal administration [62]. The absorption of reviparin was assessed by measuring the anti-FXa plasma levels and an increase in plasma anti-FXa levels to 0.4 anti-Xa U/mL was observed after administration of SNOCC at 3% (*w*/*v*) with reviparin. Recently, another chitosan derivative, *N*,*N*-dimethyl chitosan (DMC, Figure 5), was used to form a polyelectrolyte complex with UFH [63]. In vitro release studies showed that heparin is released from the complex which indicates that DMC could be used to increase the oral bioavailability of heparin. Moreover, DMC was able to efficiently protect heparin in simulated gastric fluid in which heparin is degraded. Chitosan oligomers (Figure 5) are obtained through complete deacetylation of chitosan and according to the number of sugar units in their structure they are dimer, tetramer, or hexamer. In contrast to chitosan, which has poor solubility in water at physiological pH, these chitosan oligomers are water-soluble and have relative low molecular weight. Absorption of LMWHs from the small intestine of rats was greatly increased in the presence of an optimal concentration of 0.5% (*w*/*v*) of chitosan hexamer, and the absorption of LMWH was dependent on the concentration of hexamer [64]. Biological markers such as protein and lactate dehydrogenase released from intestinal epithelial cells were measured to assess the toxicity of chitosan hexamers after intestinal administration and revealed that chitosan oligomers did not induced remarkable membrane damage to the intestine over the concentration range tested and therefore these compounds could be used as penetration enhancers with possibly few toxic effects [64].

The co-administration of LMWHs with poly(acrylate) derivative Carbopol 934P, which interferes with the intercellular junctions, was investigated. LMWH intestinal absorption was achieved with enhanced anti-FXa levels after administration of 10,800 anti-Xa U/kg LMWH with 1% (*w*/*v*) Carbopol 934P and the effect was sustained for 6 h [66].

Thiolated polymers or thiomers improve mucoadhesion properties and permeation enhancing properties due to the thiol groups [65]. The presence of thiol groups offers the advantage of forming disulfide bonds between these novel polymers and the mucus gel layer, mimicking the natural mechanism of secreted mucus glycoproteins, which are also covalently anchored in the mucus layer by the formation of disulfide bonds [60]. Polycarbophil-cystein (PCP-Cys, Figure 5) is a thiomer that was found to increase the absorption of LMWHs achieving anticoagulant levels when formulated with glutathione (GSH) [65,68]. The oral administration of a LMWH with PCP-Cys/GSH resulted in a significantly increase of LMWH absorption compared with control tablets comprising unmodified PCP or with an orally given aqueous LMWH solution, with an absolute bioavailability of ~20% [65].

Labrasol is a well-defined mixture of glycerides and fatty acids esters which was described to enhance the intestinal absorption of LMWHs [69,70]. The anti-FXa plasma levels obtained after administration of a formulation containing 200 IU/kg of LMWH and 50.0 mg/kg of labrasol was 0.50 IU/mL, which was considered adequate to exert anticoagulant activity in plasma. Significant absorption of LMWHs occurred in jejunum compared with duodenum and ileum. Intestinal membrane permeability changes induced by labrasol were transient and reversible. Later, LMWHs were dispersed with labrasol and the mixture was solidified with three different adsorbents (microporous calcium silicate, magnesium aluminometa silicate, and silicon dioxide) [71]. In a dissolution study developed in vitro, silicon dioxide system showed the fastest release rate of LMWHs among the used emulsifiers but in in vivo studies, microporous calcium silicate system showed the highest plasma anti-FXa activity (C_max_ = 0.42 ± 0.01 IU/mL) [71]. However, the mechanism by which labrasol increases LMWH intestinal absorption has not been elucidated, though it is believed to be a combination of increased solubility and enhanced permeability.

### 3.2. Transcellular Absorption

Sodium *N*-(8-[2-hydroxybenzoyl] amino) caprylate (SNAC) and sodium *N*-[10-(2-hydroxybenzoyl) amino] decanoate) (SNAD) (Figure 5) are non α-aminoacids that interact non-covalently with heparin, neutralizing its negative ionic charge to render it more lipophilic, and allowing transcellular absorption [72]. Once the complex crosses the membrane, SNAC dissociates from the therapeutic agent [52,72]. SNAC increased the absorption of heparin through the GI tract in therapeutic doses [73]. Oral heparin/SNAC entered clinical trials and showed good results in healthy volunteers and in patients undergoing elective total hip arthroplasty [74]. Oral LMWHs/SNAD also prevented deep venous thrombosis [75]. These studies demonstrated for the first time that heparins can be effectively orally delivered into the bloodstream in patients [76].

A randomized, double-blind, controlled study performed in humans to study the safety and the effect of heparin/SNAC in anticoagulation showed that heparin/SNAC increased the coagulation parameters (APTT, anti-FIIa and FXa, and tissue factor pathway inhibitor) and, that neither heparin or SNAC alone changed these parameters [77]. A major limitation seems to be heparin high first pass metabolism by heparinase in the liver after GI absorption. In a phase I clinical trial to characterize the pharmacokinetic/pharmacodynamic profile of heparin administered orally with SNAC to healthy volunteers, a higher dose of oral heparin/SNAC (75,000 IU) was necessary to achieve an anticoagulant effect similar to parenteral administration (10,000 IU) [78]. For both anti-FXa and IIa activity, oral heparin/SNAC formulation showed higher half-life time compared to intravenous injection of heparin sodium (0.666 U/mL vs. 0.0841 U/mL). Anti-FXa achieved at peak with oral heparin was 0.547 U/mL, lower than with parenteral administration for both anti-FXa and IIa activities. Area under the curve was also lower for oral heparin/SNAC than parenteral administration, for both anti-FXa and IIa activities. However, in Phase III clinical trial oral formulation of heparin/SNAC did not show superior efficacy compared to enoxaparin in patients that undergone total hip replacement [79]. Additionally, unexpected low compliance was observed due to the bad taste.

Polycationic lipophilic-core dendrons (Figure 5) have been shown to enhance absorption of LMWHs in rats [80]. After administration of LMWH (7500 IU/kg) with a polycationic lipophilic-core dendron (7 mol equivalents) a significantly increase in C_max_ was observed. These structures establish lipophilic ion-pairs with the polyanionic LMWHs making them more hydrophobic. This ion-pair model of absorption assumes that the dendrons are absorbed as a complex with LMWHs. However, poor aqueous solubility of the complex dendron-LMWHs limited their absorption. Through lipophilic ion pairing, Lee et al. designed a DOCA derivative, deoxycholylethylamine (DOCA-NH_2_) (Figure 5), to complex with LMWHs [81]. The complex was dissolved in propylene glycol and administered in rats by oral gavage. Physical association of DOCA-NH_2_ with LMWHs turned the later more lipophilic and in vivo experiments indicated that DOCA-NH_2_ significantly affected the oral absorption of the LMWHs, and at a molar ratio of 1:5 the oral absorption of LMWHs was high. An anti-FXa value of 0.83 ± 0.11 IU/mL was observed after oral administration of a 50 mg/kg dose of LMWH/DOCA-NH_2_. Nevertheless, the complex LMWHs/DOCA-NH_2_ showed reduced solubility due to its size and propylene glycol had to be used as solubilizer. The oral absorption of LMWHs/DOCA-NH_2_ complex was higher than of LMWHs/DOCA complex. The mechanism by which LMWHs absorption is improved is not clear and may be related to bile acids transporters or passive absorption. However, toxicological studies were not performed.

A number of penetration enhancers were shown to improve oral availability of heparins. In this strategy, chemical structure of heparins is preserved [82] and once the complex crosses the membrane, penetration enhancers dissociate spontaneously from the therapeutic agent [52]. However, systemic toxic side effects of these compounds cannot be excluded [83]. Consequently, mucoadhesive polymers, which are not absorbed in the GI tract due to its high molecular weight, seem more valuable compared to other penetration enhancers. In general, poor solubility of complexes formed with heparins, local and systemic toxic effects, and the fact that heparins’ degradation is not avoided, are the main drawbacks of this strategy.

## 4. Micro and Nanoparticles

Micro and nanoparticles have received considerable interest in recent years due to their ability to control the pattern of drugs’ release and due to their good pharmacokinetics.

Both polymeric micro and nanoparticles have been used to improve oral absorption of heparins [84,85,86,87,88,89,90]. Additionally, due to their unique physical and biological properties [91], nanoparticles protect heparins from pH and enzymatic degradation and are able to completely avoid the first pass metabolism by the liver.

Biodegradable polymers (poly-ε-caprolactone and poly-d,l-lactic-co-glycolic acid) and non-biodegradable polymers (Euparin dragit^®^ RS and RL) were used to prepare heparin-loaded nanoparticles [84,86,88,90]. Anticoagulant activity was maintained, but only in vitro studies were performed. These polymers were also used to prepare microparticles to encapsulate heparin [85]. Initial in vitro tests showed satisfactory encapsulation efficiency and controlled drug release with retention of the anticoagulant activity [90]. Further in vivo studies in rabbits showed absorption of heparin after oral administration [88]. The mechanism involved in the enhancement of oral bioavailability of heparin loaded polymeric nanoparticles was hypothesized to be through mucoadhesion: first there is an electrostatic interaction between nanoparticles and mucus, and after that heparin on the particle surface is released and replaced by mucin [92].

Microparticles obtained through complex coacervation of an optimized LMWH/acacia gum mixture with either gelatin A or B led to an increase of the oral bioavailability of tinzaparin [93]. Gelatin B microparticles showed higher oral bioavailability compared with gelatin A which was negligible. In vitro drug release occurred at physiological pH (pH 7.4).

Later, LMWH-loaded nanoparticles were prepared using polyester and polycationic polymethacrylate [84]. Oral absorption of this LMWH was improved and its anticoagulant effect was prolonged for up to 8 h.

Chen et al. prepared a nanoparticle system shelled with low molecular weight chitosan for oral delivery of heparin [94] that releases heparin at physiological pH. Chitosan and heparin were able to form polyelectrolyte complexes via electrostatic interaction at low pH, resulting in a matrix structure with a spherical shape with average diameters in the nano scale. A significant anticoagulant activity in plasma was observed after oral administration in rats. Intestinal absorption of heparin was significantly enhanced with a total bioavailability of 20.5%. A biodistribution study was performed and it suggested that the absorption of chitosan into the systemic circulation is minimal. Chitosan nanoparticles have been coated with an anionic polysaccharide alginate and tested as an efficient drug delivery system to achieve intestinal absorption of a LMWH (enoxaparin) [95]. Anionic polysaccharide alginate was used to overcome the problems of low solubility of chitosan at physiological pH. Alginate-coated chitosan nanoparticles loaded with enoxaparin released only 2% of the LMWH in simulated gastric fluid (pH 1.2) with faster release rate in simulated intestinal fluid conditions leading to the release of more than 60% of the LMWH. In vitro permeation studies showed that more than 75% of enoxaparin was transferred across the intestinal epithelium after 90 min. The in vivo pharmacokinetic studies showed that the alginate-coated chitosan nanoparticles significantly enhanced the oral bioavailability of enoxaparin compared with enoxaparin solution. LMWHs loaded *N*-trimethyl chitosan (TMC) nanoparticles were developed by Paliwal et al. to achieve oral bioavailability of LMWHs through mucoadhesive properties of chitosan derivatives [96]. These nanoparticles increased 2.4 times the bioavailability of LMWHs in comparison to LMWH solution.

In the literature, there is a combination of strategies through the encapsulation of heparin-lipid and heparin-DOCA conjugates in solid lipid nanoparticles [25]. Solid lipid nanoparticles consist of biodegradable physiological lipids or lipidic substances and stabilizers which are generally recognized as safe [97]. Encapsulation of this conjugate into solid lipid nanoparticles of phosphatidylcholine significantly improved the bioavailability of LMWHs after oral route administration with insignificant toxicity to different GI tissues. Histological examination of GI tract tissues after one hour of oral administration of heparin-lipid conjugate loaded solid lipid nanoparticles was performed and toxicity was not found in any part of the GI epithelium [25].

More recently, LMWHs were encapsulated in particles with polyaminomethacrylates (Eudragit^®^ RL or RS) using polyethylene glycol derivatives as solvents [98]. The use of short chain polyethylene glycol derivatives is a possible advantage due to their low toxicity when compared with other solvents and achieved nearly a complete drug release in vitro.

One advantage of micro and nanoparticles is that they protect heparins from degradation and at the same time promote heparin absorption in the GI tract. Nevertheless, this approach has drawbacks related to the delay created in drug absorption and the lack of control retained over absorption time as a result of the variability in intestinal motility and gastric emptying. Toxicity also has to be considered, since polymeric materials modify tight junctions and could lead to the absorption of endotoxins and other potentially toxic compounds [99].

## 5. Conclusions

Heparin has unique properties that are not achieved with new oral anticoagulants. The development of an orally active heparin will avoid the inconvenience of parenteral administration. In the last few years, several strategies have been attempted in order to overcome heparin’s poor bioavailability: conjugation with lipophilic molecules, co-administration with penetration enhancers, and micro and nanoparticles. While the chemical structure of heparins is preserved with penetration enhancers and the release from the therapeutic agent occurs spontaneously, dissociation before absorption is avoided by chemical conjugation. With both strategies, heparin’s degradation is not circumvented. In contrast, micro and nanoparticles protect heparins from pH and enzymatic degradation and are able to avoid the first pass metabolism. Efficient absorption with minimal toxicity has been the major concern. In fact, some of these strategies may modified tight junctions and led to systemic toxicity. Considering the use of naturally occurring substances as carriers, conjugation can correspond to a more suitable strategy. Nevertheless, none have reached the market yet. With the gathered information, we hope to assist in the rational planning of strategies to overcome heparin’s bioavailability and expect this century to be fruitful in rendering an oral active heparin or heparin-like to reach the market.

## Abbreviations

The following abbreviations are used in this manuscript:

ATIII	Antithrombin III
DMC	*N*,*N*-Dimethyl chitosan
DMSO	Dimethylsulfoxide
DOCA	Deoxycholic acid
DOCA-NH_2_	*N*-Deoxycholylethylamine
FXa	Factor Xa
GA	18β-Glycyrrhetinic acid
GI	Gastrointestinal
GSH	Glutathione
LMWHs	Low molecular weight heparins
MCC	Mono-*N*-carboxymethyl chitosan
PCP-Cys	Polycarbophil-cystein
SNAC	Sodium *N*-(8-[2-hydroxybenzoyl] amino) caprylate
SNAD	Sodium *N*-[10-(2-hydroxybenzoyl) amino] decanoate
SNOCC	*N*,*O*-carboxymethyl chitosan
UFH	Unfractioned heparin
VKA	Vitamin K antagonists
